# Crosstalk between microbiome, regulatory T cells and HCA2 orchestrates the inflammatory response in a murine psoriasis model

**DOI:** 10.3389/fimmu.2023.1038689

**Published:** 2023-02-20

**Authors:** Agatha Schwarz, Rebecca Philippsen, Serena G. Piticchio, Jan N. Hartmann, Robert Häsler, Stefan Rose-John, Thomas Schwarz

**Affiliations:** ^1^ Department of Dermatology and Allergology, University Kiel, Kiel, Germany; ^2^ Institute of Clinical Molecular Biology (IKMB), University Kiel, Kiel, Germany; ^3^ Facultat de Farmàcia, Universitat de Barcelona, Barcelona, Spain; ^4^ Institute for Biochemistry, University Kiel, Kiel, Germany

**Keywords:** G-protein-coupled receptors, imiquimod, interleukins, microbiome, psoriasis, regulatory T cells, short chain fatty acids

## Abstract

The organ-specific microbiome plays a crucial role in tissue homeostasis, among other things by inducing regulatory T cells (Treg). This applies also to the skin and in this setting short chain fatty acids (SCFA) are relevant. It was demonstrated that topical application of SCFA controls the inflammatory response in the psoriasis-like imiquimod (IMQ)-induced murine skin inflammation model. Since SCFA signal *via* HCA2, a G-protein coupled receptor, and HCA2 expression is reduced in human lesional psoriatic skin, we studied the effect of HCA2 in this model. HCA2 knock-out (HCA2-KO) mice reacted to IMQ with stronger inflammation, presumably due to an impaired function of Treg. Surprisingly, injection of Treg from HCA2-KO mice even enhanced the IMQ reaction, suggesting that in the absence of HCA2 Treg switch from a suppressive into a proinflammatory type. HCA2-KO mice differed in the composition of the skin microbiome from wild type mice. Co-housing reversed the exaggerated response to IMQ and prevented the alteration of Treg, implying that the microbiome dictates the outcome of the inflammatory reaction. The switch of Treg into a proinflammatory type in HCA2-KO mice could be a downstream phenomenon. This opens the opportunity to reduce the inflammatory tendency in psoriasis by altering the skin microbiome.

## 1 Introduction

Psoriasis is a chronic inflammatory dermatosis, with an increasing prevalence, ranging at 0.84% of the population in 2017 (1). The pathogenesis is complex involving several pathways (2). Although psoriasis is assigned to the family of autoimmune dermatoses, a definite autoantigen such as for bullous autoimmune dermatoses has not been detected (3). One pathomechanism involved appears to be a downregulation of regulatory T cells (Treg) which under normal conditions control inflammatory and immune reactions (4–6). The mechanisms responsible for this alteration of Treg are still unclear.

There is increasing evidence that the microbiome exerts profound effects on Treg, first discovered in the gut. The intestinal microbiome is able to activate Treg (7–10). These effects are partially mediated *via* short chain fatty acids (SCFA), microbiota-derived bacterial fermentation products including butyrate, propionate, and acetate (11, 12). Accordingly, certain alterations of the intestinal microbiome are associated with inflammatory bowel diseases (13–15), It is known that the intestinal microbiome can also modulate inflammatory processes of other organs like the airway system, the central nervous system and even the skin (10, 16, 17),.

Meanwhile it is clear that also other organs including the respiratory and urogenital tract and the oral cavity harbor a certain microbiome milieu which contributes to tissue homeostasis. This applies also to the skin. We demonstrated that topical application of the SCFA sodium butyrate (SB) reduced inflammation in the skin, suggesting that resident skin microbes prevent exaggerated inflammatory responses by exerting a downregulatory function and thereby maintain a stable state under physiologic conditions (18).

Furthermore, we observed that SB exerts a mitigating effect in psoriasis (19). This was primarily demonstrated in the imiquimod (IMQ)-induced inflammation model, an established murine model of psoriasis-like skin inflammation (20). Topically applied SB reduced IMQ-induced inflammation. Treg were definitely involved in this process since the mitigating effect was lost upon depletion of Treg. Furthermore, Treg isolated from the blood of psoriatic patients were reduced in their suppressive activity, which was restored by SB. Additionally, SB restored the fewer numbers of Treg in biopsies of psoriatic lesions and normalized the enhanced expression of IL-17 and IL-6 and the reduced expression of IL-10 and of Foxp3 (19).

SCFA signal *via* G-protein-coupled receptors, including GPR109a/HCA2/NIACR1 (21, 22) and GPR43 (23, 24). SB utilizes primarily HCA2 to mediate its effects. HCA2-activation in the gut exerted antiinflammatory effects, finally inducing Treg and IL-10-producing T cells (8). Accordingly, HCA2-knockout (HCA2-KO) mice showed enhanced susceptibility to colitis (21).

Due to the potential beneficial effect of SB in psoriasis, we quantified the expression of HCA2 in psoriatic skin. Lesional and non-lesional psoriatic skin revealed a decreased expression of HCA2 on keratinocytes in comparison to control skin. Topical application of SB was able to increase the low-level expression of HCA2 (25).

To get more insight into the role of HCA2 in psoriasis, we utilized the IMQ-model and HCA2-KO mice. Here, we show that HCA2-KO mice present with a remarkably increased inflammatory response to IMQ. Adoptive transfer experiments revealed that Treg in HCA2-KO mice were not only impaired in their suppressive activity, but even enhanced the inflammatory response. Obviously, Treg in the absence of HCA2 switch from a regulatory into a proinflammatory type. All these changes appear to be dictated by an altered skin microbiome as a consequence of HCA2-deficiency.

## 2 Material and methods

### 2.1 Mice

Eight to nine week old female C57BL/6J mice (WT) (Janvier Labs, stock #U03, Le Genest Saint Isle, France) and HCA2-KO (Gpr109a^-/-^) (26) mice were housed in the animal facilities of the University Clinics Schleswig-Holstein. Mice were kept under SPF conditions (individually ventilated cage, IVC, to keep an animal separated from other animals and possible exposures, including exposure by air). Water and food were autoclaved. Expert personnel in compliance with relevant laws and institutional guidelines utilized animal care. Experiments were approved by the Animal Welfare Commission of Ministry of Energy, Agriculture, the Environment, Nature and Digitalization of the Federal State Schleswig-Holstein. Animals were treated with IMQ-cream (Aldara^®^) and received 62.5 mg on the shaved backs for 7 days. For all experiments, at least 5 mice per group were used. The clinical response was quantified according to a modified psoriasis area and severity index (PASI) score (27). Skin thickness was quantified with a spring-loaded micrometer. Biopsies were paraffin embedded and sections stained with H&E. Acanthosis was semiquantitatively scored as: - <40 µm; + >40 - <60 µm; ++ >60 - <130 µm; +++ >130 µm; the inflammatory infiltrate was semiquantitatively scored as: sparse: <15 cells/10,000 µm²; moderate: >15 - <35 cells/10,000 µm²; dense: >35 cells/10,000 µm².

### 2.2 Isolation of Treg

Lymph nodes (LN) and spleens were isolated from donor animals and pooled; single cell suspensions were prepared. The proportion of CD4^+^CD25^+^ cells was more or less the same in the spleens and the LN (**Supplementary Figure 1**). CD4^+^CD25^+^ T cells were collected with a magnet-activated cell sorter (Miltenyi Biotec, Bergisch Gladbach, Germany) by using the MojoSort™ Mouse CD4^+^CD25^+^ Regulatory T Cell Isolation Kit (BioLegend, Fell, Germany, Cat #480137). CD4^+^CD25^+^ T cells were isolated in a two-step separation process. The cells were first incubated with the biotin conjugated antibody cocktail, followed by the streptavidin nanobeads, to isolate total CD4^+^ T cells. The second step consisted of a positive enrichment of CD25^+^ cells using allophycocyanin (APC) conjugated anti-mouse-CD25-antibody and anti-APC nanobeads. The purity of CD4^+^CD25^+^ cells was determined by flow analysis using rat anti-mouse-CD4 conjugated with fluorescein isothiocyanate (FITC; BioLegend; Cat #130308, RRID: AB_1279237) and rat anti-mouse-CD25 labeled with APC (Miltenyi Biotec, Cat #130-102-550, RRID: AB_2660261) and was usually more than 95%. As immunoglobulin controls rat IgG2b FITC and rat IgM APC (both from Santa Cruz Biotechnology, Dallas, USA, Cat #sc-2835, RRID: AB_737268; Cat #sc-2896, RRID: AB_737296) were used. In general, we gained up to 3% Foxp3^+^ cells of all bulk cells obtained from lymph nodes and spleens which is in accordance with the literature (28). In some experiments Treg were stained with 10 μM carboxyfluorescein succinimidylester (CFSE) (Vybrant^®^ CFDA SE Cell Tracer Kit, Molecular Probes, Oregon; Cat #V12883). After washing Treg were injected intravenously (i.v.) (1x10^6^) into recipient animals.

### 2.3 Suppression assay

1x10^6^ cells/ml CD4^+^CD25^-^ responder T cells obtained from WT mice were seeded into 96-well plates and mixed with CD4^+^CD25^+^ Treg obtained from WT or HCA2-KO mice at the ratios of 1:1, 2:1 and 4:1. Responder T cells were activated using anti-CD3 and anti-CD28-bound Dynabeads (Dynabeads Mouse T-Activator CD3/CD28, Life technologies, Carlsbad, USA). After 4 days, cell proliferation was measured using Cell Counting Kit-8 (Sigma-Aldrich, Taufkirchen, Germany). Data are presented as percentage of suppression.

### 2.4 IL-6 experiments

HCA2-KO mice were injected intraperitonealy (i.p.) once with a rat monoclonal anti-IL-6-antibody (clone MP5-20F3; 270 µg; Abcam, Cambridge, England; Cat #ab191194). On day 7 after injection, Treg were isolated from untreated or anti-IL-6-treated HCA2-KO mice and injected i.v. into WT mice.

WT mice were injected i.p. daily for 7 days with 1 µg human recombinant IL-6 expressed in *E. coli* and purified to homogeneity as described (29). We used human IL-6, since it acts both on human and murine cells (30). On day 8 Treg were isolated and injected i.v. into WT animals.

The serum from WT and HCA2-KO mice was collected and the amount of IL-6 was measured using the LEGEND MAX Mouse IL-6 ELISA Kit (BioLegend; #Cat 431307) according to the manufacturer’s instructions. Serum samples were assayed in triplicates and absorbance read using the TECAN Infinite M Plex (TECAN, Männedorf, Switzerland) microplate reader at 450 nm and 560 nm.

### 2.5 Flow cytometry

For intracellular cytokine staining, cell suspension was prepared from pooled LN and spleens and stimulated with PMA and Ionomycin in the presence of Brefeldin A (Cell Activation Cocktail with Brefeldin A; BioLegend; Cat #423303) for 3 hours. After stimulation cells were fixed and permeabilized (Tru-Nuclear Transcription Factor, BioLegend, Cat #424401). Cells were stained for APC-conjugated anti-mouse-Foxp3 (eBioscience, San Diego, USA; Cat #17-5773-82, RRID: AB_469457) and phycoerythrin (PE) conjugated anti-mouse-IL-10 (Cat #505007, RRID: AB_315361, -GARP (Cat #142904, RRID: AB_10962944), -CD25 (Cat #102007, RRID: AB_312857) and -IL-6-antibodies (Cat #504503, RRID: AB_315337; (BioLegend). Furthermore, the following antibodies were used: PE-conjugated anti-mouse CTLA-4 (Cat #106305, RRID: AB_313254, Ki-67 (Cat #151209, RRID: AB_2716014), PD-1 (Cat #109103, RRID: AB_313420), CD73 (Cat #127205, RRID: AB_1089065), FR4 (Cat #125007, RRID: AB_1134202), IL-17A (Cat #506903, RRID: AB_315463), IL-23 (Cat #505203, RRID: AB_315367) and Alexa-488 conjugated anti-mouse Helios (Cat #137213, RRID: AB_10645334; all from BioLegend).

FACS analysis of CFSE-negative and positive cells was conducted using APC-conjugated anti-mouse-IL-6- (Cat # 504507, RRID: AB_10694094), -IL-17A- (Cat #506915, RRID: AB_536017), -IL-23- (Cat # 505205, RRID: AB_315369) and -IL-10- antibodies (Cat # 505009, RRID: AB_315363; all from BioLegend).

Flow analysis after co-housing experiments was performed using rat anti mouse CD4-APC (Miltenyi Biotec, Cat #130-102-597, RRID: AB_2659906) and rat anti mouse Foxp3-PE (eBioscience, Cat #12-5773-80, RRID: AB_465935). As isotype controls the following immunoglobulins: rat IgG2a-APC (Cat #sc-2894, RRID: AB_737246) as control for Foxp3-APC; rat IgG2b-APC (Cat #sc-2895, RRID: AB_737266) as control for IL-10-APC and CD4-APC (both from Santa Cruz); rat IgG2a-PE (Becton Dickinson; Cat #553930, RRID: AB_479719) as control for GARP-PE and Foxp3-PE; rat IgG2b-PE (Cat #556925, RRID: AB_479625) as control for IL-10-PE; rat IgG1-APC (Cat #400411, RRID: AB_326517) as control for IL-6-, IL-17-, IL-23-APC; rat IgG1-PE (Cat #400407, RRID: AB_326513) as control for IL-6-, CD25-, IL-17- and IL-23-PE (both from BioLegend); rat IgG2b-PE (Cat # 556925, RRID: AB_479625) as control for PD-1 and Ki-67-PE (Becton Dickinson); armenian hamster IgG1-PE (Becton Dickinson; Cat #553972, RRID: AB_395172) and Alexa-488 conjugated (BioLegend; Cat #400923; RRID: AB_2814703) as control for CTLA-4-PE, and Helios-A488; rat IgG1-PE (Miltenyi Biotec, Cat # 130-123-746, RRID: AB_2857627) as control for CD73- and FR4-PE. Analysis was performed with a CytoFlex (Beckman Coulter).

### 2.6 16S rRNA sequencing

Animals were wiped off with wet swabs in both directions (head to tail and tail to head). To generate representative bacteria based on relative abundancies, 16S sequencing was performed as previously described (31). DNA was extracted using the QIAamp UCP Pathogen Mini Kit automated on the QIAcube (Qiagen, Hilden, Germany) following the manufacturer’s guidelines.

16S rRNA gene libraries were generated by PCR from purified genomic DNA with primers 27F and 338R, targeting the hypervariable regions V1 and V2 of the 16S rRNA. Amplification and sequencing were performed using a dual-indexing approach (8-nt on forward- and reverse primer) as described by Kozich et al. (32) on the Illumina MiSeq platform (Illumina Inc., San Diego, USA) generating 2x300 bp reads. Demultiplexing after sequencing was based on no mismatches in the indices.

Data processing was performed using the DADA2 version 1.10 workflow for big data sets (33) resulting in abundance tables of amplicon sequence variants (ASVs) according to a workflow adjusted for V1-V2 region, which can be found here: https://github.com/mruehlemann/ikmb_amplicon_processing/blob/master/dada2_16S_workflow_with_AR.R. Resulting ASVs underwent taxonomic annotation using the Bayesian classifier provided in DADA2 and using the Ribosomal Database Project (RDP) version 16 release. One sample with less than 10,000 sequences was not considered for further analysis. ASVs classified as “Chloroplast” were removed. Alpha diversity was estimated with Shannon index and Beta diversity was estimated from Bray-Curtis dissimilarity (*phyloseq* R package). Differential abundance analysis was performed with the linear model function LinDA (34) in the *MicrobiomeStat* package, which can be found at https://CRAN.R-project.org/package=MicrobiomeStat.

### 2.7 Statistical analysis

In all experiments except FACS analysis and 16S rRNA sequencing results were analyzed by using the Student t test. For FACS analysis t test with Welch’s correction was performed. For multiple comparisons we carried out one-way ANOVA in order to assess whether at least two groups significantly differ. Differences between groups were determined with unpaired two-samples Wilcoxon test for alpha diversity, and PERMANOVA (*adonis2* function of *vegan* R package) for beta diversity. Linear models included confounders (housing, genotype) where applicable. For all analysis p-values <0.05 were considered significant.

## 3 Results

### 3.1 HCA2-deficiency enhances the susceptibility to psoriasis-like skin inflammation

**Figure 1:**
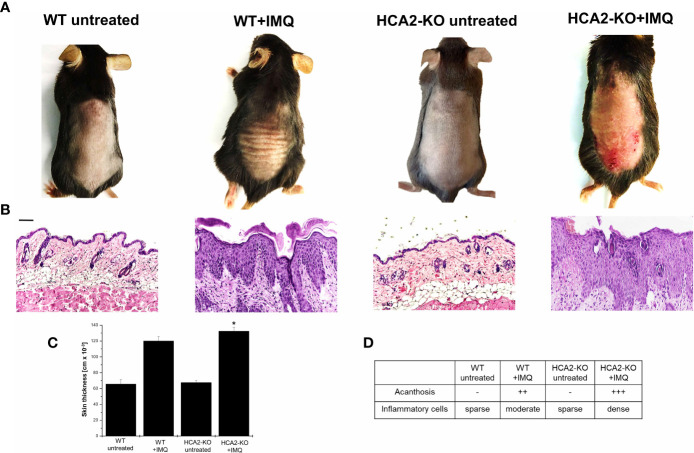
HCA2 deficiency enhances the susceptibility to psoriasis-like skin inflammation. **(A)** WT and HCA2-KO mice were treated topically with 5% IMQ cream on the shaved backs for 7 (d) **(B)** Biopsies were taken, paraffin embedded sections were stained with H&E and analyzed histopathologically. Each group contained 7 animals. Scale bar = 100 µm **(C)** Skin thickness was measured using a spring-loaded micrometer. Student t test and one-way ANOVA was performed. *P < 0.02 WT+IMQ vs HCA2-KO+IMQ; P_ANOVA_ = 1.9 x10^-10^ **(D)** Acanthosis and density of inflammatory infiltrate were scored. Data are presented from one of three independent experiments (n=3).

To study the role of HCA2 in psoriasis and its impact on Treg, we utilized the IMQ-induced inflammation model and mice which are deficient in the expression of HCA2. C57BL/6J wild type (WT) and HCA2-KO mice received IMQ-cream on the shaved backs daily for 7 days. As described previously (19), IMQ-treated mice developed thickening of the skin, erythema and scales. In HCA2-KO animals the inflammatory response was remarkably stronger (**Figure 1A**). The clinical response was quantified for this and all following experiments according to a modified PASI score (27). The data of all experiments are summarized in **Supplementary Figure 2**. Accordingly, measurement of skin thickness revealed swollen skin upon administration of IMQ which was maximally pronounced in IMQ-treated HCA2-KO mice (**Figure 1C**).

Biopsies were taken and sections stained with H&E. IMQ-treatment induced acanthosis, hyperkeratosis and an inflammatory infiltrate. These changes were stronger pronounced in HCA2-KO mice in comparison to WT animals (**Figures 1B, D**). Since IMQ, though only topically applied, induces also systemic alterations (20), mice were sacrificed 7 days after initiation of IMQ treatment and spleens were obtained. Spleens of IMQ-treated animals were enlarged and heavier (19). Splenomegaly was stronger pronounced in HCA2-KO mice upon application of IMQ (data not shown).

### 3.2 HCA2 signaling regulates the suppressive activity and the phenotype of Treg

**Figure 2:**
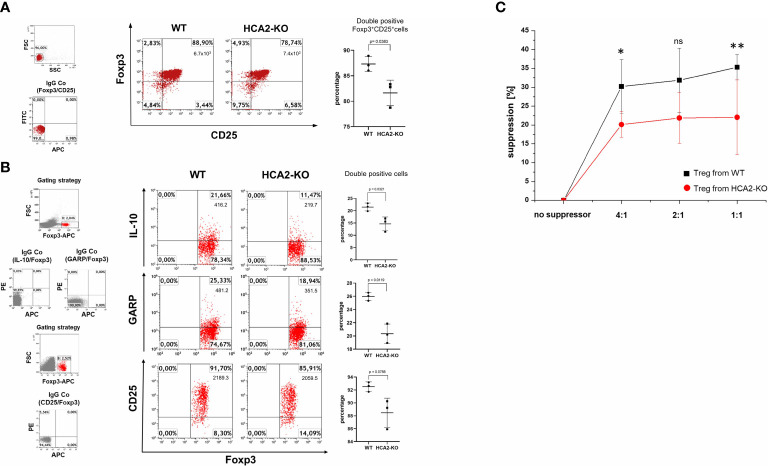
HCA2 signaling regulates the suppressive activity *in vitro* and the phenotype of Treg. **(A)** CD4^+^CD25^+^ (Treg) cells were isolated from pooled spleens and lymph nodes (LN) of 7 mice and subjected to FACS analysis. The cell population from both strains was analyzed for double positive CD25/Foxp3 cells and is demonstrated as percentage and total number of double positive cells. As negative control served isotype (IgG Co). FACS analysis is also shown as a scatter graph with mean ± SD. Y axes show percentage of double positive cells. Data were analyzed by using the t test with Welch´s correction. n = 3 **(B)** Pooled LN cells and splenocytes of 4 mice, obtained from both strains were analyzed for double positive Foxp3/IL-10, Foxp3/GARP and Foxp3/CD25 cells and is demonstrated as percentage and total number of double positive cells. FACS analysis is also shown as a scatter graph with mean ± SD. Y axes show percentage of double positive cells. Data were analyzed by using the t test with Welch´s correction. n = 3 Data are presented from one of three independent experiments. **(C)** Pooled LN and spleen cells obtained from WT and HCA2-KO mice (4 for each group) were separated into CD4**^+^** CD25^-^ (responder cells) and CD4^+^CD25^+^ cells (Treg). Treg and responder cells were mixed at the ratios 1:1, 1:2 and 1:4. Responder cells were activated and after 4 days, cell proliferation was measured using Cell Counting Kit-8. Data are presented as percent suppression from one of three independent experiments. Student t test and one-way ANOVA was performed. *P < 0.03 WT vs HCA2-KO 4:1; P = 0.11504 WT vs HCA2-KO 2:1; ns, not significant; **P < 0.04 WT vs HCA2-KO 1:1; P_ANOVA_ = 0.016; n = 4.

We previously observed that IMQ-induced inflammation is associated with a decrease/suppression of Treg (19). To analyze whether the enhanced susceptibility of HCA-KO mice to IMQ is due to an impairment of Treg, adoptive transfer experiments were performed. Spleens and LN were isolated from HCA2-KO or WT mice and pooled. Cell suspensions from pooled organs were subjected to FACS analysis. HCA2-KO mice harbored reduced percentage of CD4^+^CD25^+^Foxp3^+^ cells (**Figure 2A**) and also the expression of CD25 on Foxp3^+^ cells was impaired (**Figure 2B**). Foxp3^+^ cells from HCA-KO mice expressed lower levels of IL-10 and glycoprotein A repetitions predominant (GARP), which is specifically expressed on activated Treg (35) (**Figure 2B**).

To better define Treg obtained from HCA2-KO mice, more markers which are associated with the suppressive activity of Treg were analyzed. Foxp3^+^Helios^+^Treg exert more suppressive characteristics compared to Foxp3^+^Helios^-^ Treg (36). Ki-67 is an activating marker for Treg (37). The ecto-5’-nucleosidase (CD73) contributes to the inhibitory function of Treg by generating adenosine (38). The folate receptor 4 (FR4) is required for the activity of Treg (39). The expression of CTLA-4, CD73, FR4, Helios, Ki-67 was reduced on Foxp3^+^ cells obtained from HCA2-KO mice in comparison to WT cells (**Supplementary Figure 3**). Furthermore, the expression of PD-1 which inhibits activation and suppressive capacity of Treg (40, 41) was upregulated in Foxp3^+^ cells from HCA2-KO mice (**Supplementary Figure 3**).

To analyze whether this phenotypic alteration is also associated with a functional impact, the suppressive activity of Treg obtained from WT and HCA2-KO mice was evaluated in a suppression assay. CD4^+^CD25^-^ responder cells were coincubated at different ratios with Treg obtained either from WT or HCA2-KO mice and proliferation was measured. The proliferation of CD4^+^CD25^–^ responder cells was reduced upon coincubation with Treg obtained from WT mice. This effect was reduced upon coincubation with Treg obtained from HCA2-KO mice (**Figure 2C**).

**Figure 3:**
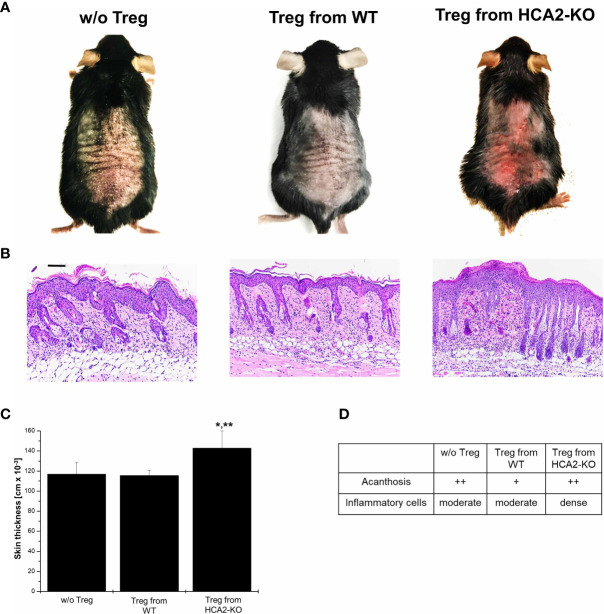
HCA2 signaling regulates the suppressive activity *in vivo* **(A)** Treg were isolated from pooled spleens and LN of WT or HCA2-KO mice and injected i.v. into IMQ-treated WT animals. **(B)** Biopsies obtained from IMQ-treated animals were taken. The paraffin embedded sections were stained with H&E and analyzed histopathologically. Each group contained 7 animals. Scale bar = 100 µm **(C)** The skin thickness was measured using a spring-loaded micrometer. Student t test and one-way ANOVA was performed. *P < 0.02 Treg from WT vs Treg from HCA2-KO, **P < 0.02 Treg from HCA2-KO vs w/o Treg; P_ANOVA_ = 0.00872. **(D)** Acanthosis and density of inflammatory infiltrate were scored. Data are presented from one of three independent experiments.

To examine whether the altered phenotype of HCA2-KO Treg also influences their suppressive potency *in vivo*, CD4^+^CD25^+^ cells were isolated from pooled spleens and LN of WT and HCA2-KO mice, respectively. The purity was in the range at least of 95%. Cells were injected i.v. into WT animals which subsequently were treated with IMQ. Injection of Treg obtained from WT mice mitigated IMQ-induced inflammation in WT recipients (**Figure 3A**). Down-modulation of inflammation by Treg was confirmed histopathologically (**Figures 3B, D**). Surprisingly, the inflammatory response to IMQ in mice which received Treg from HCA-KO mice was not at all reduced but even remarkably enhanced (**Figure 3A**). Skin thickness analysis confirmed this observation (**Figure 3C**). This suggests that HCA2-deficient Treg are not only impaired in their suppressive activity but even enhance inflammation. This phenomenon may contribute to the enhancement of the “psoriatic” response in the absence of HCA2.

### 3.3 HCA2-deficiency causes defects of Treg at the site of inflammation

**Figure 4:**
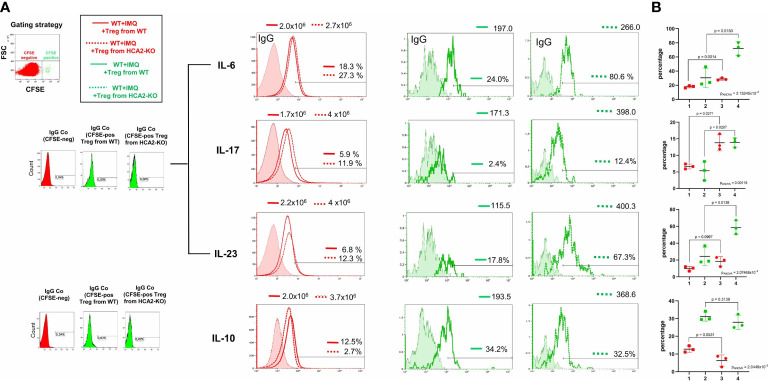
HCA2 deficiency causes defects of Treg at the site of inflammation. **(A)** Treg obtained from 7 HCA2-KO and 7 WT mice were stained with CFSE and injected i.v. into IMQ-treated WT animals. After 48 h LN and spleens were obtained from the recipient mice and FACS analysis of CFSE-negative (overlay of red histograms) or CFSE-positive cells (overlay of green histograms) was conducted. The expression of IL-6, IL-17, IL-23 and IL-10 was standardized on isotype controls. For green histograms (Treg from WT and HCA2-KO) two isotype controls were used. For IL-6, IL-17 and IL-23 the same IgG (rat IgG1) control was used. Histograms show fluorescence intensity (x axis) versus cell count (y-axis). The number of cells is displayed in the histograms. **(B)** FACS analysis is also shown as scatter graph with mean ± SD. Y axes show percentage of positive cells. Data were analyzed by using the t test with Welch´s correction. For multiple comparisons we carried out one-way ANOVA in order to assess whether at least two groups significantly differ. 1 WT +IMQ + Treg from WT (red); 2 WT +IMQ + Treg from WT (green); 3 WT +IMQ + Treg from HCA2-KO (red); 4 WT +IMQ + Treg from HCA2-KO (green) n=3.

Under normal conditions, Treg suppress inflammation, but several publications demonstrated a dysfunction of Treg, particularly in autoimmune diseases (42). To study whether this might also apply for IMQ-induced psoriasis-like inflammation, we stained Treg obtained from WT and HCA2-KO mice with CFSE. Fluorescent Treg were injected i.v. into IMQ-treated WT animals. After 48 hours LN and spleens were obtained from the recipients and FACS analysis of CFSE-negative cells was conducted. This strategy allows to detect the inflammatory status of host cells after injection of Treg. IMQ upregulated the proinflammatory cytokines IL-6, IL-17, and IL-23 in comparison to untreated WT mice (**Supplementary Figure 4**). The expression of these cytokines was reduced in host cells (CFSE-negative) upon injection of WT-Treg. In contrast, such an effect was not observed upon injection of Treg from HCA2-KO mice. In this case, the expression of the proinflammatory cytokines was even enhanced in the host cells. In turn, the expression of the “suppressive” cytokine IL-10 was induced upon injection of Treg from WT mice in the host cells, but impaired upon injection of Treg from HCA2-KO mice (**Figure 4**; overlay of red histograms). Statistical analysis was performed after having pooled the data from 3 experiments (**Figure 4B**).

To gain insight into the production of cytokines by the adoptively transferred cells (CFSE-positive), flow analysis was performed by gating on CFSE^+^ cells. FACS analysis revealed that injected Treg obtained from HCA2-KO animals expressed enhanced levels of IL-6, IL-17, and IL-23 in comparison to the expression of these cytokines in WT-Treg (**Figure 4A**; overlay of green histograms). IL-10 was only slightly downregulated in injected Treg from HCA2-KO mice compared to WT Treg. However, those differences were statistically not significant. Statistical analysis was performed after having pooled the data from 3 experiments (**Figure 4B**). A limitation of this experiment is the low cell number recovered from the recipients. However, this was the maximum yield achieved when injecting 1x10^6^ cells per mouse. Nevertheless, the alteration of the cytokine expression pattern in the recipients upon injection of Treg from different donors appears to correspond to the clinical phenotype (**Figure 3A**).

### 3.4 IL-6 is involved in the alteration of Treg in the absence of HCA2

**Figure 5:**
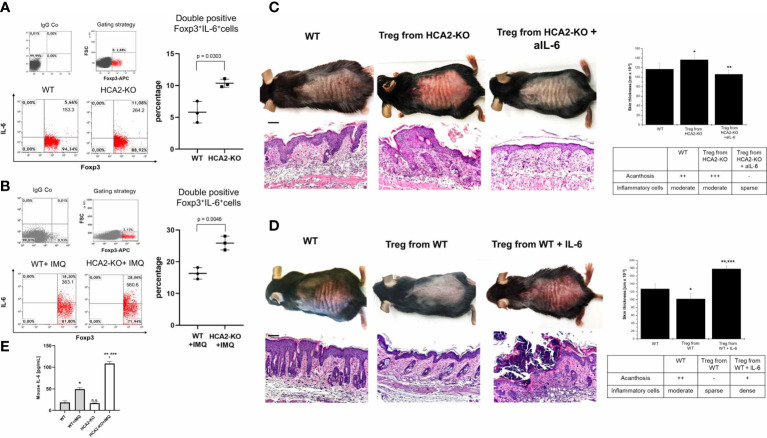
IL-6 appears to be involved in the alteration of Treg in the absence of HCA2. Pooled LN cells and splenocytes obtained from 4 naïve mice **(A)** or 4 IMQ-treated **(B)** WT and HCA2-KO mice were analyzed for double positive Foxp3/IL-6 cells and is demonstrated as percentage and total number of double positive cells. FACS analysis is also shown as a scatter graph with mean ± SD. Y axes show percentage of double positive cells. Data were analyzed by using the t test with Welch´s correction. n = 3 **(C)** Treg were obtained from naïve HCA2-KO or from HCA2-KO mice injected i.p. with a rat monoclonal anti-IL-6 antibody. The isolated Treg cells were injected i.v. into IMQ-treated WT mice. Biopsies from IMQ-treated recipients were taken; paraffin embedded sections were stained with H&E and analyzed histopathologically. Scale bar = 100µm. Skin thickness was measured using a spring-loaded micrometer. Student t test and one-way ANOVA was performed. *P < 0.025 WT vs Treg from HCA2-KO, **P < 0.0003 Treg from HCA2-KO vs Treg from HCA2-KO+anti IL-6; P_ANOVA_ = 9.9x10^-4^. Acanthosis and density of inflammatory infiltrate were scored. Each group contained 7 animals. **(D)** Treg were obtained from naïve WT mice or WT mice injected i.p. with IL-6. The isolated Treg cells were injected i.v. into IMQ-treated WT mice. Biopsies from IMQ-treated recipients were taken; paraffin embedded sections were stained with H&E and analyzed histopathologically. Macroscopical and histopathologic pictures of untreated WT mice are not shown for the sake of space. Scale bar = 100 µm. Skin thickness was measured using a spring-loaded micrometer. Student t test and one-way ANOVA was performed. *P < 0.02 WT vs Treg from WT, **P < 0.0002 WT vs Treg from WT+IL-6, ***P < 0.002 Treg from WT vs Treg from WT+IL-6; P_ANOVA_ = 3.2x10^-5^. Acanthosis and density of inflammatory infiltrate were scored. Each group contained 7 animals. Data are presented from one of three independent experiments. **(E)** Serum was obtained from untreated or IMQ-treated WT and HCA2-KO mice, respectively, and IL-6 serum levels were measured using an ELISA. Data were analyzed by using the t test with Welch´s correction and one-way ANOVA. *P = 0.0004 WT vs WT+IMQ; **P = 0.0008 HCA2-KO vs HCA2-KO+IMQ; ***P = 0.0001 WT+IMQ vs HCA2-KO+IMQ; n.s. = 0.4098 WT vs HCA2-KO; P_ANOVA_ = 3.05815x10^-11^; n = 3.

Since IL-6 participates in the pathogenesis of inflammatory and autoimmune diseases by downregulating Treg (43, 44), we studied whether IL-6 plays a role in the alteration of Treg in the absence of HCA2. FACS analysis of Treg revealed higher expression of IL-6 in HCA2-KO than in WT cells (**Figure 5A**). Epicutaneous application of IMQ induced the expression of IL-6 in Treg in both groups but to a much higher extent in HCA2-KO mice (**Figure 5B**). To address whether IL-6 is relevant for the functional alteration of Treg, HCA2-KO donors were injected i.p. with a neutralizing monoclonal anti-IL-6-antibody or left untreated. Treg were isolated from both groups and injected i.v. into IMQ-treated WT mice. The inflammatory response was significantly mitigated in the recipients of Treg obtained from anti-IL-6-treated HCA2-KO mice compared to recipients of Treg from untreated HCA2-KO donors (**Figure 5C**). Histopathological analysis revealed reduced inflammatory and epidermal effects in the skin of recipients of cells from anti-IL-6-treated HCA2-KO donors. We have not yet analyzed the phenotypic alteration of Treg obtained from HCA2-KO mice upon anti-IL-6 treatment. Preliminary data indicate that in the recipients of cells of anti-IL-6-treated donors Treg express enhanced levels of IL-10 and GARP which may contribute to the mitigation of the IMQ-induced inflammatory response (**Supplementary Figure 5**). In turn, WT Treg lost their suppressive activity upon injection of IL-6 into the donors. In this case the inflammatory IMQ-response was not at all reduced in the recipients upon adoptive transfer of Treg (**Figure 5D**). In addition, IL-6 serum levels were increased upon IMQ administration, this increase was much more pronounced in HCA2-KO than WT mice (**Figure 5E**).

### 3.5 Skin dysbiosis in the absence of HCA2 may predispose mice to psoriasis-like inflammation

The skin and mucosal surfaces are colonized by large numbers of microorganisms commonly referred to as the microbiota. A complex interplay between the host immune system and the microbiota is necessary to maintain homeostasis in the gut and the skin (45, 46). Accordingly, interruptions of the relationship between the host and the microbiota can cause autoimmune reactions (47). Since there is a crosstalk between HCAs and the microbiota which involves also SCFA, we studied whether HCA2-KO mice differed in their skin microbiota from WT mice. Skin swab sampling of the back skin was performed. The microbiome of HCA2-KO mice housed under regular conditions in our animal facility was characterized by 16S rRNA sequencing and compared to that of WT mice. We hypothesized that the impaired activity of Treg and the thereby caused enhanced susceptibility to psoriasis-like inflammation upon IMQ-treatment could be explained by the transmission of a disease-predisposing bacterial community. Therefore, we performed co-housing experiments with WT and HCA2-KO mice. WT mice were co-housed with HCA2-KO mice for 1 month. For control purposes strains were housed in a single setting.

**Figure 6:**
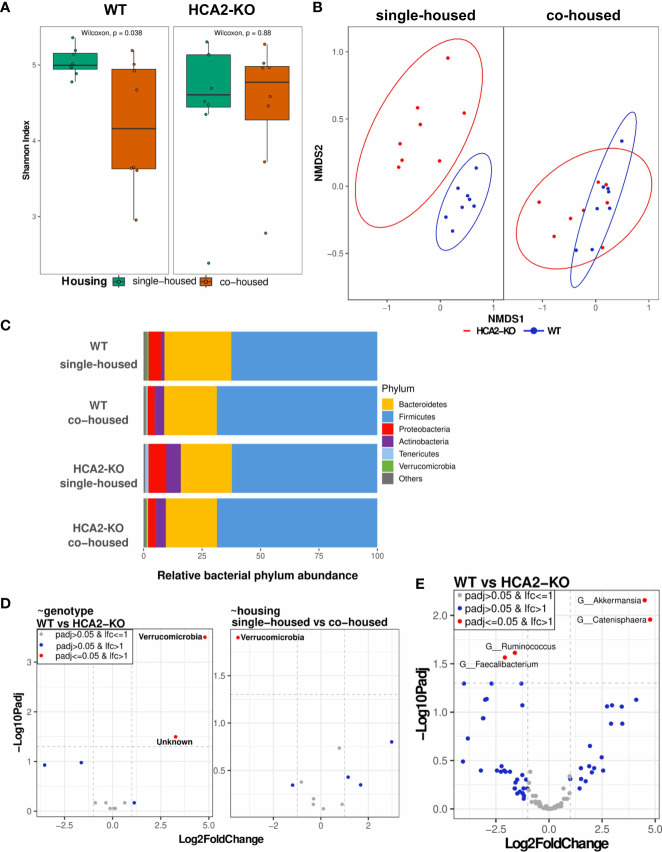
Skin dysbiosis in the absence of HCA2 predisposes mice to psoriasis-like inflammation. WT mice were co-housed with HCA2-KO mice for 1 month. 16S rRNA sequencing was performed. **(A)** Alpha diversity (Shannon Index) between WT and HCA2-KO mice, while colors indicate housing conditions. **(B)** Beta diversity calculated with Bray-Curtis dissimilarity and displayed by non-metric multidimensional scaling (NMDS) between single- and co-housed mice, while colors indicate genotype. **(C)** Relative bacterial abundance at phylum level split for the different genotype and housing conditions. **(D)** Differential abundance at phylum level based on linear models by genotype (left) and by housing (right). Volcano plot visualizes –Log10 Benjamini-Hochberg adjusted p-value over Log2FoldChange (LFC). LFC is depicted as relative to HCA2-KO (left) and co-housed (right), i.e. a negative LFC displaying a reduced abundance in WT compared to HCA-KO mice in the left graph and a reduced abundance in single-housed compared to co-housed mice in the right graph. **(E)** Differential abundance at genus level based on linear models by genotype. Volcano plot visualizes –Log10 Benjamini-Hochberg adjusted p-value over Log2FoldChange. A negative LFC shows genera with reduced abundance in WT mice, whereas a positive LFC shows genera more abundant in WT mice. No genus was significantly differentially abundant by housing (data not shown). Each group contained 8 animals. Data are presented from one of three independent experiments.

The skin microbiome of mice, as a key immunomodulatory player, was analyzed based on swab samples, assessing composition and diversity employing 16S rRNA sequencing methods. A decrease in alpha diversity (Shannon index, quantifying within sample variation) was observed upon co-housing the animals. This effect was more pronounced in WT mice (P = 0.038), while in HCA2-KO mice the observed trend was not significant (**Figure 6A**). Similarly, assessing the beta diversity (Bray-Curtis dissimilarity, quantifying between sample variation) documented that WT animals and HCA2-KO mice represent to separate groups based on their microbiome when single-housed (P = 0.001), while their differences were reduced when co-housed (**Figure 6B**). Analysis of the relative abundance of the phyla revealed a higher proportion of *Bacteroidetes* and *Verrucomicrobia* in WT animals than in HCA2-KO mice when single-housed.

In contrast to that, single-housed HCA2-KO mice showed higher proportions of *Proteobacteria*, *Actinobacteria* and *Tenericutes* than WT animals (**Figure 6C**). In co-housed conditions, these differences were diminished. A detailed differential abundance analysis based on linear models uncovered *Verrucomicrobia* as the only significantly regulated phylum, showing a higher abundance when comparing WT animals versus HCA2-KO mice (p-value (P) = 0.003, Log2FoldChange (LFC) = 4.8) as well as when comparing co-housed vs. single housed scenarios (P = 0.013, LFC = 3.5; **Figure 6D**). As part of this, *Verrucomicrobia* could not be detected in any of the single housed HCA2-KO mice. Further characterization of differentially abundant genera revealed significantly reduced *Ruminococcus* (P = 0.024, LFC –1,6) and *Faecalibacterium* (P = 0.027, LFC -2.1) genus abundances in WT compared to HCA2-KO mice, whereas *Akkermansia* (P = 0.007, LFC 4.5) and *Catenisphaera* (P = 0.011, LFC 4.8) genus abundances were significantly elevated in WT compared to HCA2-KO mice (**Figure 6E**).

**Figure 7:**
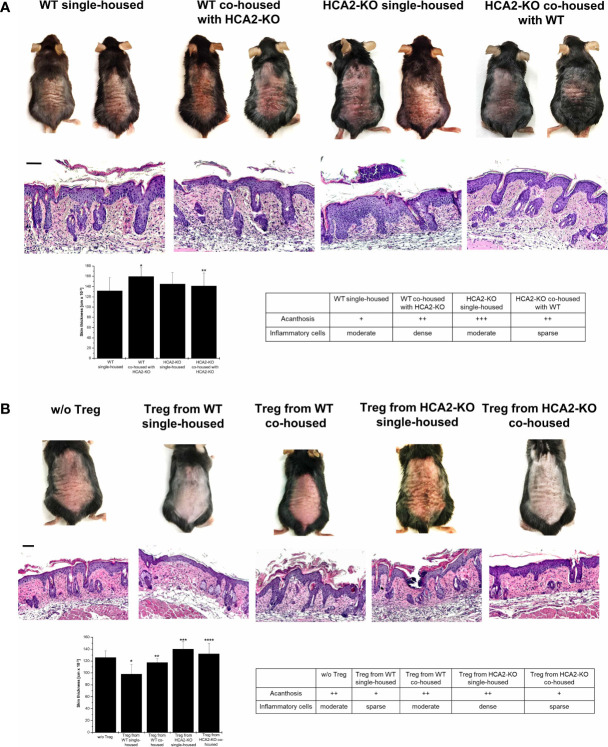
The skin microbiota determines the suppressive activity and function of Treg in HCA2-KO mice. **(A)** WT mice were co-housed with HCA2-KO mice for 1 month (each group contained 6 animals). Mice were treated topically with 5% IMQ cream on the shaved backs for 7 days. Biopsies were taken, paraffin embedded sections stained with H&E and analyzed histopathologically. Single housed HCA2-KO and WT mice served as controls. Scale bar = 100 µm. The skin thickness was measured using a spring-loaded micrometer. Student t test and one-way ANOVA was performed. *P < 0.015 WT single-housed vs WT co-housed, **P < 0.04 HCA2-KO single-housed vs HCA2-KO co-housed; P_ANOVA_ = 0.00758. The acanthosis and space of inflammatory cells was scored. Data are presented from one of three independent experiments. **(B)** WT mice were co-housed with HCA2-KO mice for 1 month. Treg were obtained either from 6 co-housed HCA2-KO or 6 co-housed WT mice and injected i.v. into IMQ-treated WT mice. As controls, Treg from 6 single-housed HCA2-KO or 6 single-housed WT mice were injected in the same fashion. Biopsies were taken, paraffin embedded sections stained with H&E and analyzed histopathologically. Scale bar = 100 µm. Skin thickness was measured using a spring-loaded micrometer. Student t test and one-way ANOVA were performed. *P < 0.009 w/o Treg vs Treg from WT single-housed, **P < 0.015 Treg from WT single-housed vs Treg from WT co-housed, ***P < 0.0025 Treg WT single-housed vs Treg from HCA2-KO single-housed, ****P < 0.05 Treg from HCA2-KO single-housed vs from HCA2-KO co-housed; P_ANOVA_ = 0.00113. Acanthosis and density of inflammatory infiltrate were scored. Data are presented from one of three independent experiments.

These changes gave rise to the speculation that the susceptibility to IMQ-induced psoriasis-like inflammation in HCA2-KO mice may be due to a dysbiotic bacterial flora which ultimately alters the function of Treg. Thus, we studied whether the altered skin microbiota is associated with the outcome of IMQ-induced inflammation in single- and co-housed mice. Therefore, animals were treated for 7 days with IMQ after 1 month of co-housing. As described above, the psoriasis-like skin inflammation was enhanced in single-housed HCA2-KO animals, when compared to single-housed WT mice. The morbidity (skin redness, scaling, hemorrhage and thickness) was significantly enhanced in WT mice upon co-housing with HCA2-KO animals (**Figure 7A**). This was also observed histopathologically. In contrast, the exaggerated inflammatory response of IMQ-treated HCA2-KO animals co-housed with WT mice was remarkably mitigated (**Figure 7A**). Together, these changes of the skin microbiota might be responsible for increased susceptibility to psoriasis-like skin inflammation in HCA2-KO animals.

### 3.6 The skin microbiota may determine the suppressive activity and function of Treg in HCA2-KO mice

Given that the transmissible, modified composition of skin microbiota might be responsible for the increased susceptibility to psoriasis-like skin inflammation in HCA2-KO mice, we hypothesized that a further consequence of the microbiota shift might be an altered activity of Treg. This was addressed by adoptive transfer experiments under co-housing conditions. After 1 month of co-housing Treg were isolated from all groups and injected into WT mice which were treated with IMQ. Treg obtained from single-housed WT mice reduced IMQ-induced inflammation. In contrast, Treg from WT mice co-housed with HCA2-KO mice did not mitigate the IMQ-induced inflammatory response (**Figure 7B**). The reverse was observed for HCA2-KO mice. As described above, the inflammatory response was significantly stronger pronounced in animals injected with Treg from single-housed HCA2-KO mice. Upon injection of Treg from co-housed HCA2-KO mice, the inflammatory response was not enhanced, as observed upon injection of Treg from single housed HCA2-KO donors, but even reduced. The macroscopic alterations were also reflected by histopathologic analysis of representative skin samples (**Figure 7B**).

## 4 Discussion

While the impact of the microbiome on the pathophysiology of psoriasis has been demonstrated in several studies (48), the mechanism by which the microbiome can modulate inflammatory responses in psoriasis remains mostly unclear. Here, we suggest how alteration of the microbiome can impact the inflammatory response in psoriasis. Dysbiosis of the skin microbiome may shift Treg from a suppressive into a proinflammatory type. Expression and signaling, respectively, of the G-protein-coupled receptor HCA2 appears to be crucially involved.

The rationale for this study was several-fold. It was primarily based on the observation that the SCFA SB can mitigate the inflammatory response in the IMQ-model which is accepted as a suitable model for psoriasis-like skin inflammation (20). There is also evidence that the mitigating effect of SB might also apply to the human system (19). Since SB signals *via* the G-protein-coupled receptor HCA2 and HCA2 expression is reduced in lesional psoriatic skin (25), we were interested to study the effect of HCA2 deficiency in the IMQ-model.

The inflammatory response to IMQ was much stronger pronounced in HCA2-KO mice. This exaggeration was not only confined to the skin but appeared to be also systemic as IMQ-induced splenomegaly was much stronger pronounced in HCA2-KO than in WT mice (data not shown). Since one pathogenetic mechanism in psoriasis is a reduced activity of Treg (4–6), we analyzed whether the same applies to HCA2 deficiency. Adoptive transfer experiments, however, yielded the surprising result that Treg from HCA2-KO mice were not only impaired in their suppressive activity but even enhanced the inflammatory response. Preliminary *in situ* double immunofluorescence analysis for Foxp3 and CD25 revealed a lower number of double positive cells in the skin of mice which had received Treg from HCA2-KO mice in comparison to recipients from Treg of WT donors. Upon injection of Treg from HCA2-KO mice, IMQ-treated WT recipients expressed higher levels of IL-17, IL-23 and IL-6 in comparison to the expression of these cytokines in WT-recipients of Treg from WT mice. In contrast, the expression of IL-10 was reduced upon injection of Treg from HCA2–KO mice. We have not analyzed whether the Treg express skin-homing receptors and thus cannot dissect at this stage the contribution of resident Treg versus into the skin immigrating Treg to the modulation of the cutaneous inflammation.

A similar alteration was previously described for several autoimmune disorders which were associated with pathogenic T helper-17 (Th17) cells (49) as well as with dysfunctional Treg (50). Bovenschen et al. (51) demonstrated that psoriatic Treg exhibit a tendency to decrease the expression of the master regulator Foxp3 and develop towards IL-17-producing Treg. Thus, our results imply that Treg in the absence of HCA2 might switch from a regulatory into a proinflammatory type. Soler et al. (52) identified IL-23 as the cytokine primarily responsible for this conversion (51). In concordance with this, we observed that upon inflammatory condition the expression of IL-17 and IL-23 was increased in HCA2-KO mice (**Supplementary Figure 6**). Furthermore, upon injection of HCA2-KO Treg, the recipients expressed higher levels of IL-23 in comparison to recipients of WT-Treg (**Figure 4**). The purity of CD4^+^CD25^+^ cells after positive selection was around 95%. This population, however, contained also a minor fraction of Foxp3-negative cells. Due to the intracellular expression, selection of Foxp3-positive cells for further functional analysis is not possible; which is a limitation of the method. However, since the proinflammatory shift was associated with a decrease of Foxp3 expression, we assume that the CD4^+^CD25^+^Foxp3^+^ cells are mostly responsible for this alteration.

This shift appears to be mediated *via* IL-6 since injection of a neutralizing anti-IL-6-antibody into HCA2-KO donors prevented such an alteration of Treg. In turn, a similar modification of Treg was observed when IL-6 was injected into WT donors. In this case, Treg enhanced the IMQ-response upon adoptive transfer. The mechanism for the enhanced expression of IL-6 in the absence of HCA2 remain to be determined as well as the mechanism by which IL-6 alters the status of Treg. In the latter scenario, histone deacetylation appears to play a role. Bovenschen et al. (51) demonstrated that in psoriasis Foxp3^+^ Treg convert into IL-17-Treg. The histone deacetylase inhibitor trichostatin-A blocked this conversion. Accordingly, we observed that histone acetylation in Treg obtained from psoriasis patients was decreased compared to H3 histones from healthy controls (19). SB upregulated histone acetylation, implying that SB acts as a histone deacetylation inhibitor (18). Whether IL-6 acts as a histone deacetylation inhibitor remains to be determined. Furthermore, IL-6 serum levels were increased upon IMQ administration. This increase was much more pronounced in HCA2-KO than WT mice.

The modification of Treg in the absence of HCA2 could be influenced by the skin microbiome. Since there is a crosstalk between HCAs and the microbiota which involves also SCFA (12, 21, 25, 53–57), we studied the impact of HCA2 on the skin microbiota by comparing WT animals to HCA2-KO mice employing 16S rRNA sequencing of skin swab samples.

Primarily, we observed a microbiome dysbiosis in susceptible animals, similar to what has been described previously for psoriasis as well as for other inflammatory diseases affecting epithelia in humans (58). The exact nature of this dysbiosis is difficult to disentangle, as the bacterial communities often consist of hundreds of species. Nevertheless, in the present scenario, we observed a substantially elevated relative abundance of the genus *Akkermansia*, from the phylum *Verrucomicrobia* in WT mice when compared to the HCA2-KO animals (**Figure 6E**). Of note, it is known that *Akkermansia (A.) muciniphila* has a protective effect against inflammation because *A. muciniphila* ameliorates chronic colitis of mice through the cross-talk of microbe-derived SCFAs and Foxp3^+^ Treg (59, 60). Furthermore, *A. muciniphila* supplementation suppressed colon inflammation and increased the frequency of colonic Treg (61). Additionally, previous studies reported that *p.Verrucomicrobia* is negatively correlated with obesity, since reduced abundance of *p*.*Verrucomicrobia* in the gut resulted in obesity in humans and animals (62, 63). HCA2-KO mice also tend to obesity. Interestingly, the majority of publications analyzed the role of *p.Verrucomicrobia* only in the gut. One of the reasons seems to be that *Akkermansia* was initially described as a strict anaerobe. However, more recently, it was reported that *Akkermansia* tolerates small amounts of oxygen and even benefits from low levels of oxygen (64). Along this line, *Verrucomicrobia* was also detected on the skin. Stehlikova et al. (65) analyzed the microbiota of the skin and of the intestine in Balb/c and C57BL/6 mice and detected also a low amount of *Verrucomicrobia* on the skin. It might be possible that the skin microbiota was not very often searched for *Verrucomicrobia*, because it was anticipated that this phylum is strictly anaerobe.

In our experiments, the microbiome displayed at least partially a causative component. When mice interacted, e.g. by being co-housed, their microbiome was transferred between each other. In the experimental setup presented here, this resulted in the microbiomes becoming more similar to each other. This microbial transfer resulted in transferring phenotypic features. We observed that co-housed HCA2-KO mice showed slightly less inflammation in the IMQ-model than single-housed HCA2-KO mice. This difference seems to be maintained when administering the respective Treg. While we observed that WT mice have an increased inflammatory phenotype in the IMQ-model when co-housed with HCA2-KO mice, our experimental setup does not allow to identify the specific species responsible for this difference. Besides this limitation, it is probable that this is due to the ensemble of additional species that populate WT animals when co-housed with HCA2-KO mice, as observed in the analysis of alpha and beta diversity. This dysbiosis could cause a hyperstimulation of the immune system resulting in increased inflammation. In this context, it has to be noted that our data does not allow to pinpoint a direct molecular effect on the host, such as correlations between alpha diversity and molecular markers (e.g., SCFA receptor mRNA expression). Following this up would require a separate study with an experimental design that specifically aims to elucidate host-microbiome interactions. Couturier-Maillard et al. (66) reported that disturbances of balance state of commensals in the gut directly or indirectly contribute to the pathogenesis of several immune-mediated intestinal illnesses such a Crohn’s disease and colitis. They observed that WT mice that were transiently co-housed with Nod2-KO mice, which are more susceptible to an induced colitis, developed an increased susceptibility to colitis. In concordance with this, we demonstrated similar effects in the IMQ-induced psoriasis-like skin inflammation model. We extended co-housing for up to two months. At this point, the phenotypic alterations remained more or less the same. The same seems to apply to the microbiota.

As a final point, it should be mentioned that *A*. *muciniphila* represents a gut microbiota signature in psoriasis (67). 16S rRNA sequencing revealed that the abundance of *A. muciniphila* was significantly reduced in patients with psoriasis. *A. muciniphila* is believed to have an important function in the pathogenesis of inflammatory bowel diseases and obesity. The authors reported that beside an alteration in the total diversity of microbiota between psoriasis and healthy control, the abundance of *A. muciniphila* is remarkably reduced in patients with psoriasis. This does not only support the validity of the model employed here, but also indicates a potential key-role of *Akkermansia* in this crosstalk, which still requires further studies. These should be focusing on species level classification by advanced methods like metagenomics, additional PCR screening and metabolomics studies to infer causality on molecular level.

Keeping this in mind, our data still illustrates changes in the relative composition of the HCA2-KO skin microbiome. While little is known about skin microbiota producing SCFA, our data cannot exclude either origin of the SCFA. Further studies will be needed to determine whether SCFA are produced in the skin at significant levels to alter an immune response. Nevertheless, topical application of SB was found to tone down inflammatory responses (18). However, we have not yet tested whether topically applied SB reduces the exaggerated IMQ response in HCA2-KO mice.

**Figure 8:**
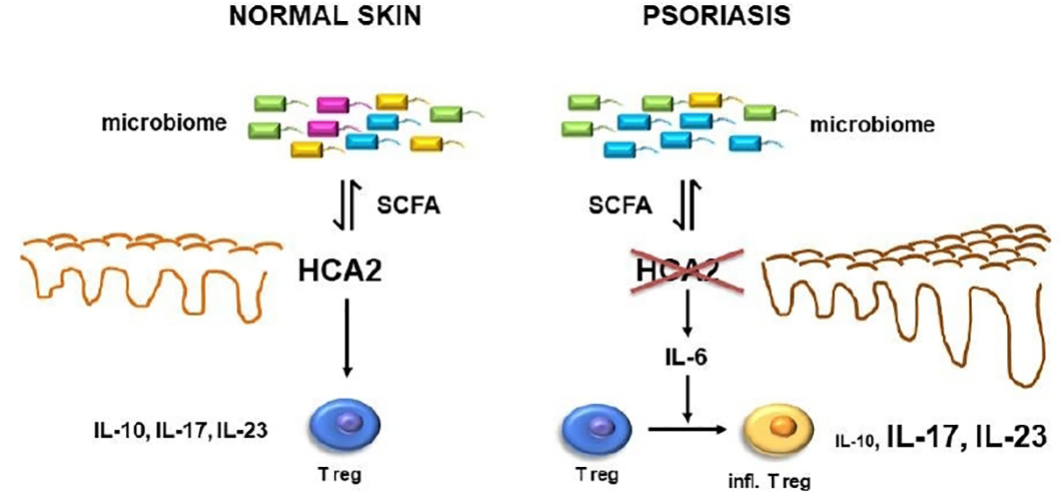
In psoriasis, reduced expression of HCA2 which may be mutually influenced by an altered skin microbiome may result in enhanced expression of IL-6 which may switch Treg into a proinflammatory cell type. This appears to be associated with a reduced expression of IL-10 and an enhanced expression of IL-17 and IL-23.

The data so far demonstrated the consequences of HCA2-deficiency several-fold: (i) the psoriasis-like inflammation response to IMQ is remarkably enhanced; (ii) Treg may switch from a regulatory into a proinflammatory type; (iii) IL-6 expression is enhanced; (iv) the skin microbiome is altered, which appears to be the most upstream phenomenon in this cascade (**Figure 8**). This was supported by co-housing experiments. The regulatory activity of Treg in WT mice could be impaired by co-housing of WT mice with HCA2-KO mice, and in turn the proinflammatory activity of Treg in HCA2-KO mice could be reversed by co-housing with WT mice. We have not yet performed detailed phenotypic analysis of the Treg within the co-housing experiments, preliminary data indicate that the enhanced proinflammatory activity appears to be associated with a decreased expression of Foxp3 and vice versa. Since the co-housing was associated with an alteration of the skin microbiome, we surmise that this could be the most upstream event. The *in vivo* response to IMQ was changed accordingly upon co-housing. However, our co-housing experiments cannot rule out changes in the microbiome of the gut or any other organ as well which is a limitation of this *in vivo* model.

While causality of the microbiome and the corresponding molecular effects on the host site are difficult to demonstrate, our findings provide further support for the hypothesis that the microbiome may play a key role in modulating the host’s immune response *via* HCA2 and Treg in the murine psoriasis-like inflammation model. These findings may have implications for the development of future strategies to treat psoriasis. Firstly, the altered skin microbiome could be changed with the ultimate aim to modulate the inflammatory psoriatic response, as it has been already demonstrated successfully for atopic dermatitis (68, 69). A second option would be topical application of SB. The effect may be several-fold, firstly a direct modulation of Treg, as demonstrated previously (16), and secondly *via* restoration of the expression of HCA2 (25) which ultimately influences the skin microbiome.

## Data availability statement

The datasets presented in this study can be found in online repositories. The names of the repository/repositories and accession number(s) can be found below: https://www.ncbi.nlm.nih.gov/, PRJNA836403.

## Ethics statement

The animal study was reviewed and approved by Animal Welfare Commission of Ministry of Energy, Agriculture, the Environment, Nature and Digitalization of the Federal State Schleswig-Holstein.

## Author contributions

Conceptualization: AS and TS; Formal Analysis: AS, RH, SR-J, JH, and SP; Funding Acquisition: AS and TS; Investigation: AS and RP; Methodology: AS, RH, SR-J, JH, and SP; Resources: TS and SR-J; Supervision: AS and TS; Validation: AS, RH, SP, and JH; Visualization: AS, RP, and JH; Writing - Original Draft Preparation: AS, RH, and TS; Writing - Review and Editing: TS. All authors contributed to the article and approved the submitted version.

## Supplementary material

Supplementary Figure 1 Cells from LN and spleens obtained from 4 WT and 4 HCA2-KO mice were isolated separately and subjected to FACS analysis. Cells were gated for Foxp3 and double positive cells (Foxp3/CD25) were analyzed. The number of double positive cells is displayed in the histograms. FACS analysis is also shown as scatter graph with mean ± SD. Y axes show percentage of double positive cells. Data were analyzed by using the t test with Welch´s correction (n = 3).

Supplementary Figure 2 The clinical response to IMQ was quantified according to a modified PASI score by Wang et al. (24). The data for **Figures 1A**, **3A**, **5C, D**, **7A, B** are shown.

Supplementary Figure 3 Pooled lymph node cells and splenocytes obtained from 4 WT and 4 HCA2-KO mice were gated for Foxp3 and analyzed for the expression of CTLA-4, Helios, Ki-67, PD-1, CD73, FR4. The total cell number of double positive cells is displayed in the histograms. FACS analysis is also shown as scatter graph with mean ± SD. Y axes show percentage of double positive cells. Data were analyzed by using the t test with Welch´s correction (n = 3).

Supplementary Figure 4
**(A)** Treg obtained from 7 HCA2-KO and 7 WT mice were stained with CFSE and injected i.v. into IMQ-treated WT animals. After 48 h LN and spleens were obtained from the recipient mice and FACS analysis of CFSE-negative (overlay of red histograms) was conducted. The expression of IL-6, IL-17, IL-23 and IL-10 was standardized to isotype controls. For IL-6, IL-17 and IL-23 the same IgG (rat IgG1) control was used. Histograms show fluorescence intensity (x axis) versus cell count (y-axis). The number of total cells is displayed in the histograms. **(B)** FACS analysis is also shown as scatter graph with mean ± SD. Y axes show percentage of double positive cells. Data were analyzed by using the t test with Welch´s correction. 1 WT untreated; 2 WT +IMQ + w/o Treg (n = 3).

Supplementary Figure 5 Treg were obtained from naïve HCA2-KO or from HCA2-KO mice injected i.p. with a rat monoclonal anti-IL-6 antibody. The isolated Treg were injected i.v. into IMQ-treated WT recipient mice. LN and spleen cells from these recipients were analyzed for double positive Foxp3/IL-10, Foxp3/GARP and Foxp3/CD25 cells and demonstrated as percentage and total number of double positive cells.

Supplementary Figure 6 Pooled lymph node cells and splenocytes were obtained from 4 IMQ-treated WT and 4 HCA2-KO mice. Cells were gated for Foxp3 and FACS analysis performed for CD25, IL-17 and IL-23. The number of double positive cells is displayed in the histograms. FACS analysis is also shown as scatter graph with mean ± SD. Y axes show percentage of double positive cells. Data were analyzed by using the t test with Welch´s correction (n = 3).
