# Supplementary figures and images for: Crosstalk between microbiome, regulatory T cells and HCA2 orchestrates the inflammatory response in a murine psoriasis model
Source: Front Immunol. 2023 Feb 20;14:1038689. doi: 10.3389/fimmu.2023.1038689 (PMC9986334; doi:10.3389/fimmu.2023.1038689)

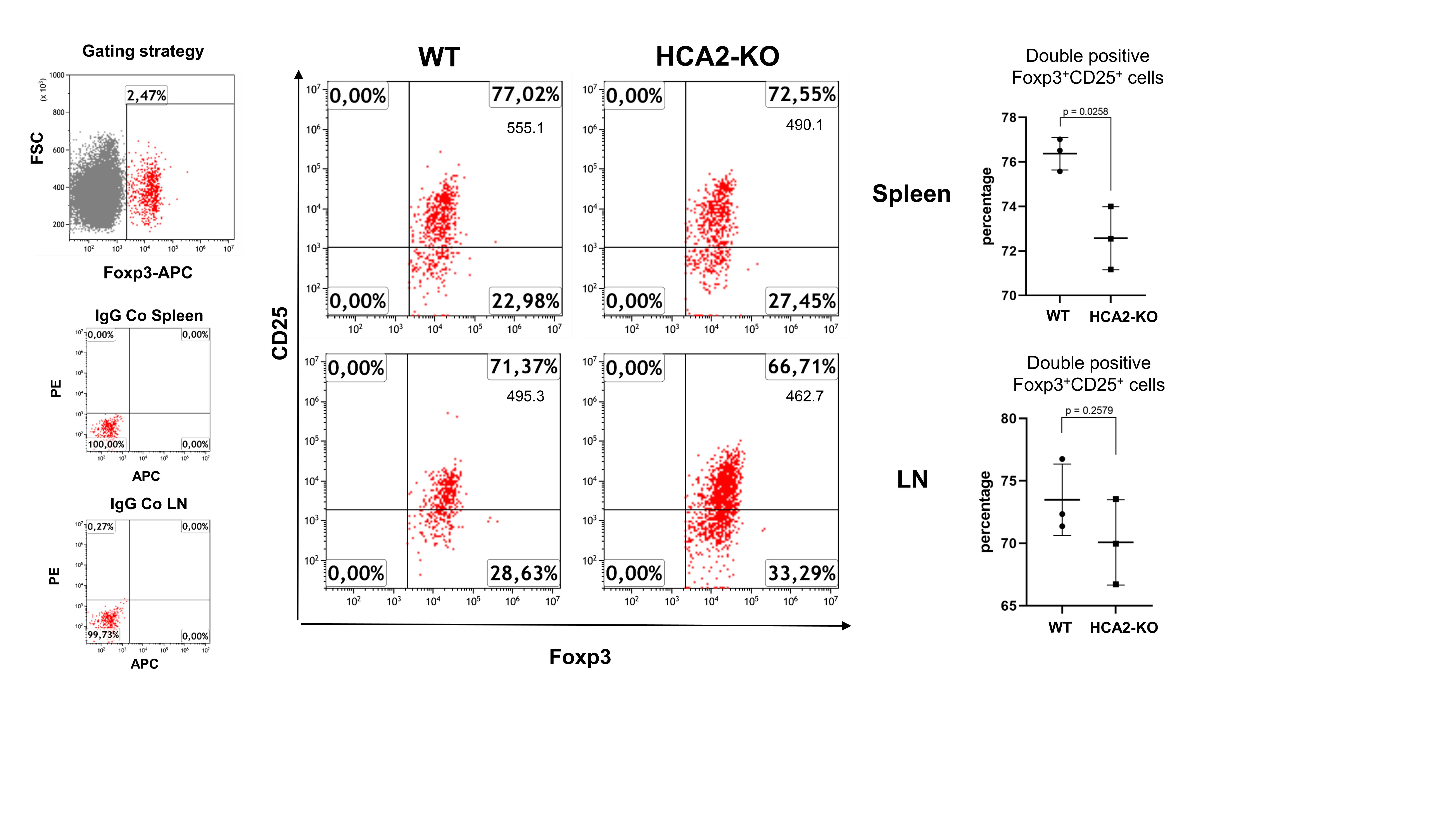

Supplement: Supplementary Figure 1 — Cells from LN and spleens obtained from 4 WT and 4 HCA2-KO mice were isolated separately and subjected to FACS analysis. Cells were gated for Foxp3 and double positive cells (Foxp3/CD25) were analyzed. The number of double positive cells is displayed in the histograms. FACS analysis is also shown as scatter graph with mean ± SD. Y axes show percentage of double positive cells. Data were analyzed by using the t test with Welch´s correction (n = 3). [file Image_1.jpeg]

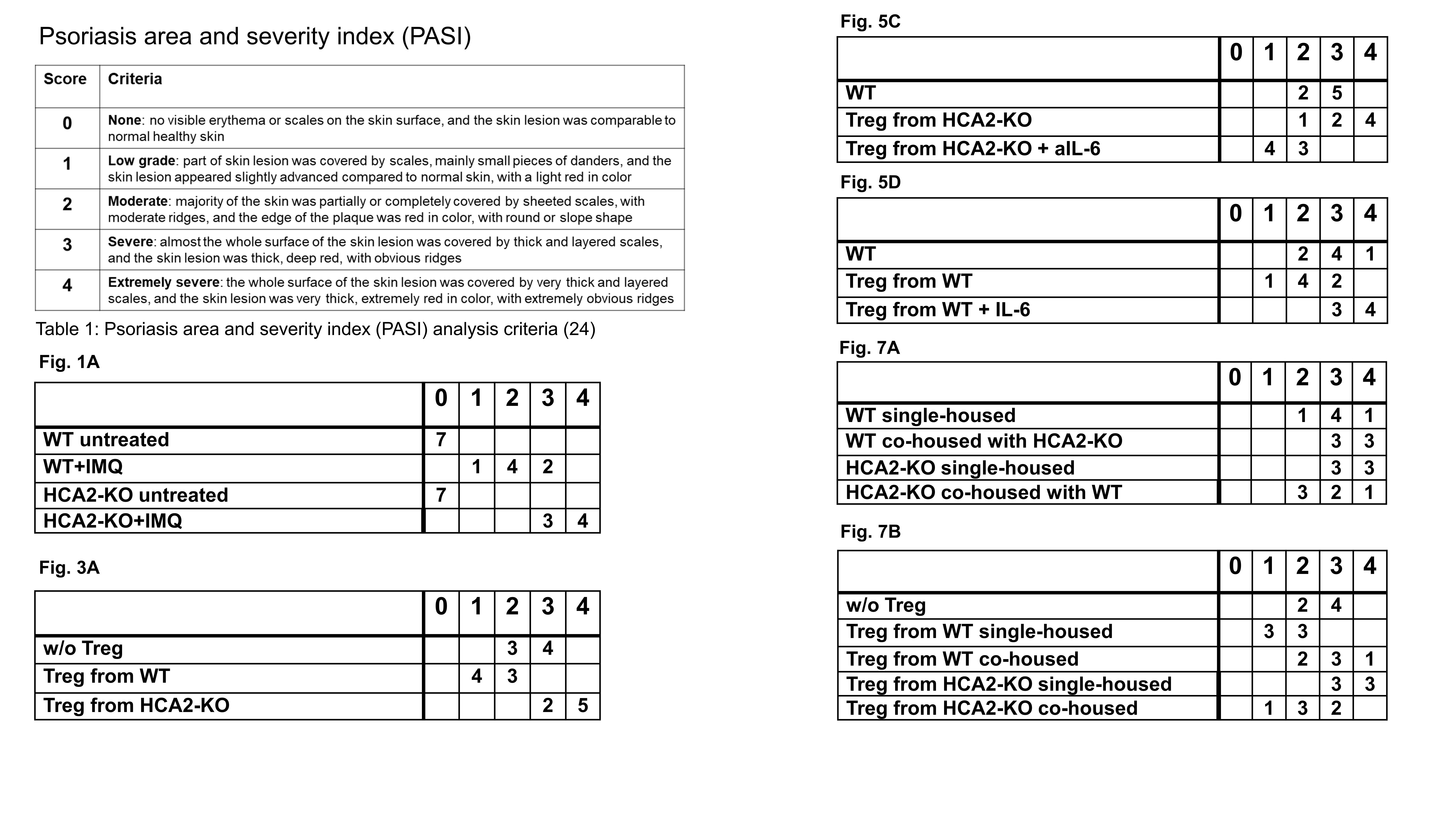

Supplement: Supplementary Figure 2 — The clinical response to IMQ was quantified according to a modified PASI score by Wang et al. (24). The data for Figures 1A , 3A , 5C, D , 7A, B are shown. [file Image_2.jpeg]

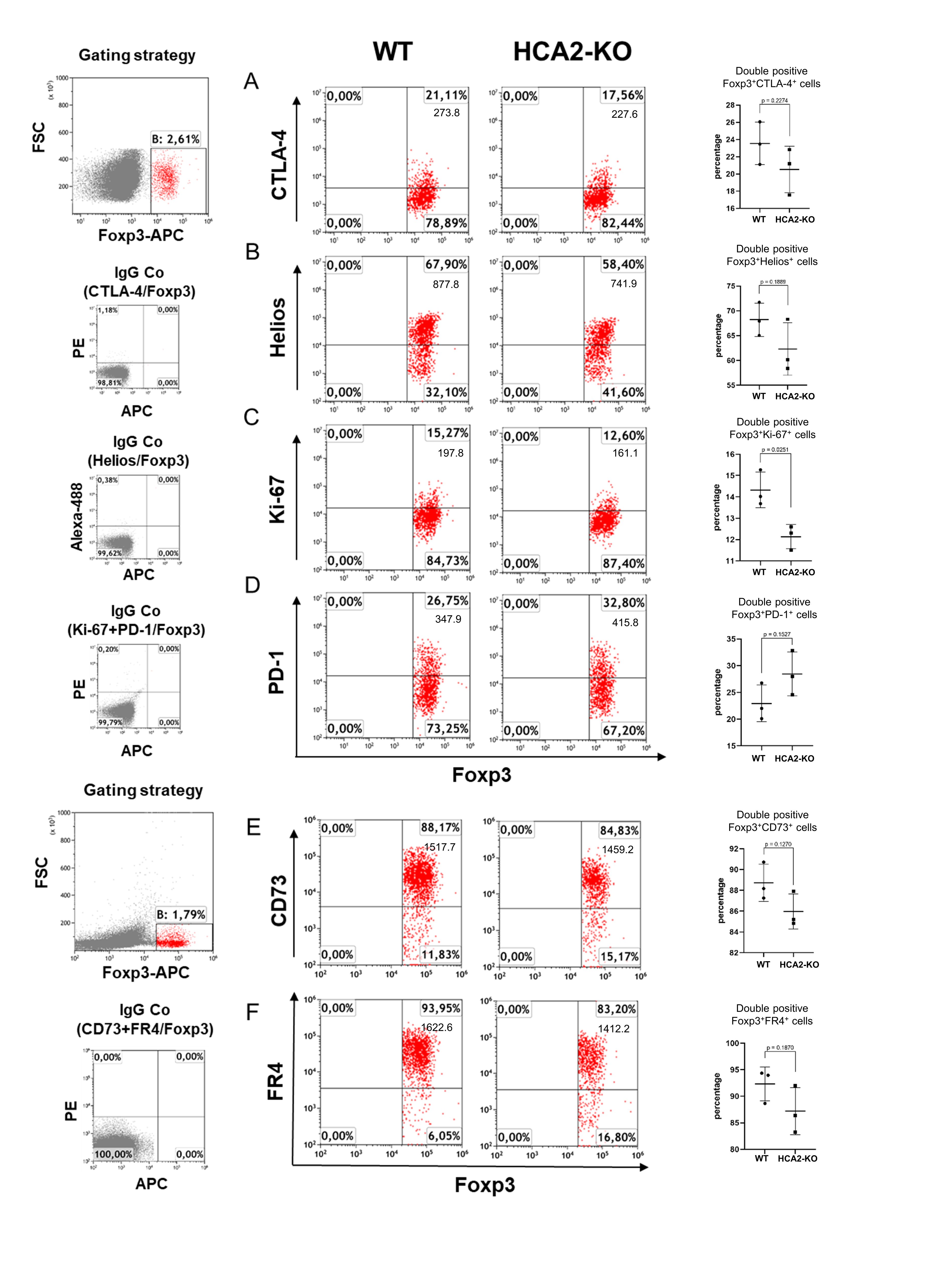

Supplement: Supplementary Figure 3 — Pooled lymph node cells and splenocytes obtained from 4 WT and 4 HCA2-KO mice were gated for Foxp3 and analyzed for the expression of CTLA-4, Helios, Ki-67, PD-1, CD73, FR4. The total cell number of double positive cells is displayed in the histograms. FACS analysis is also shown as scatter graph with mean ± SD. Y axes show percentage of double positive cells. Data were analyzed by using the t test with Welch´s correction (n = 3). [file Image_3.jpeg]

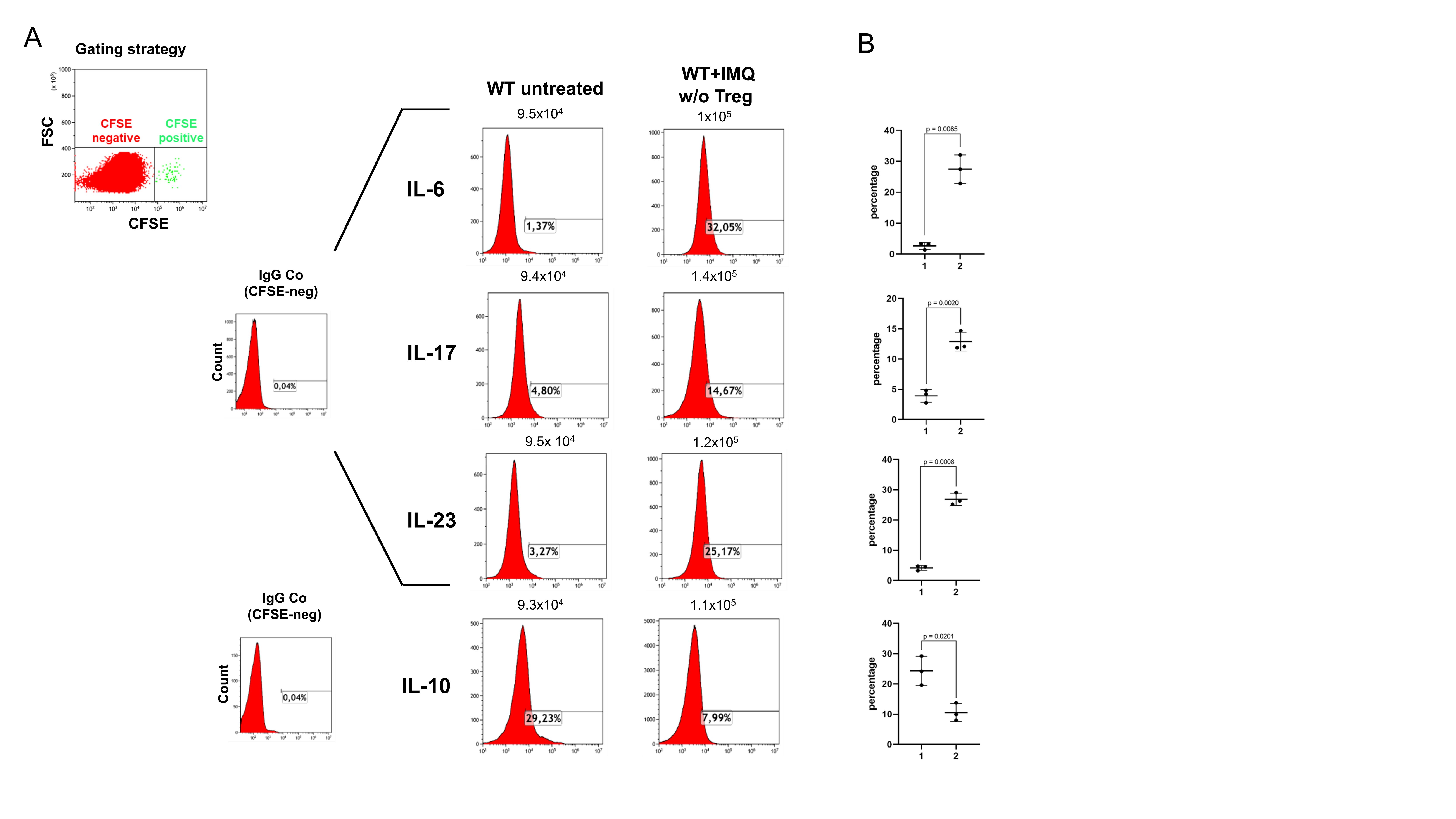

Supplement: Supplementary Figure 4 — (A) Treg obtained from 7 HCA2-KO and 7 WT mice were stained with CFSE and injected i.v. into IMQ-treated WT animals. After 48 h LN and spleens were obtained from the recipient mice and FACS analysis of CFSE-negative (overlay of red histograms) was conducted. The expression of IL-6, IL-17, IL-23 and IL-10 was standardized to isotype controls. For IL-6, IL-17 and IL-23 the same IgG (rat IgG1) control was used. Histograms show fluorescence intensity (x axis) versus cell count (y-axis). The number of total cells is displayed in the histograms. (B) FACS analysis is also shown as scatter graph with mean ± SD. Y axes show percentage of double positive cells. Data were analyzed by using the t test with Welch´s correction. 1 WT untreated; 2 WT +IMQ + w/o Treg (n = 3). [file Image_4.jpeg]

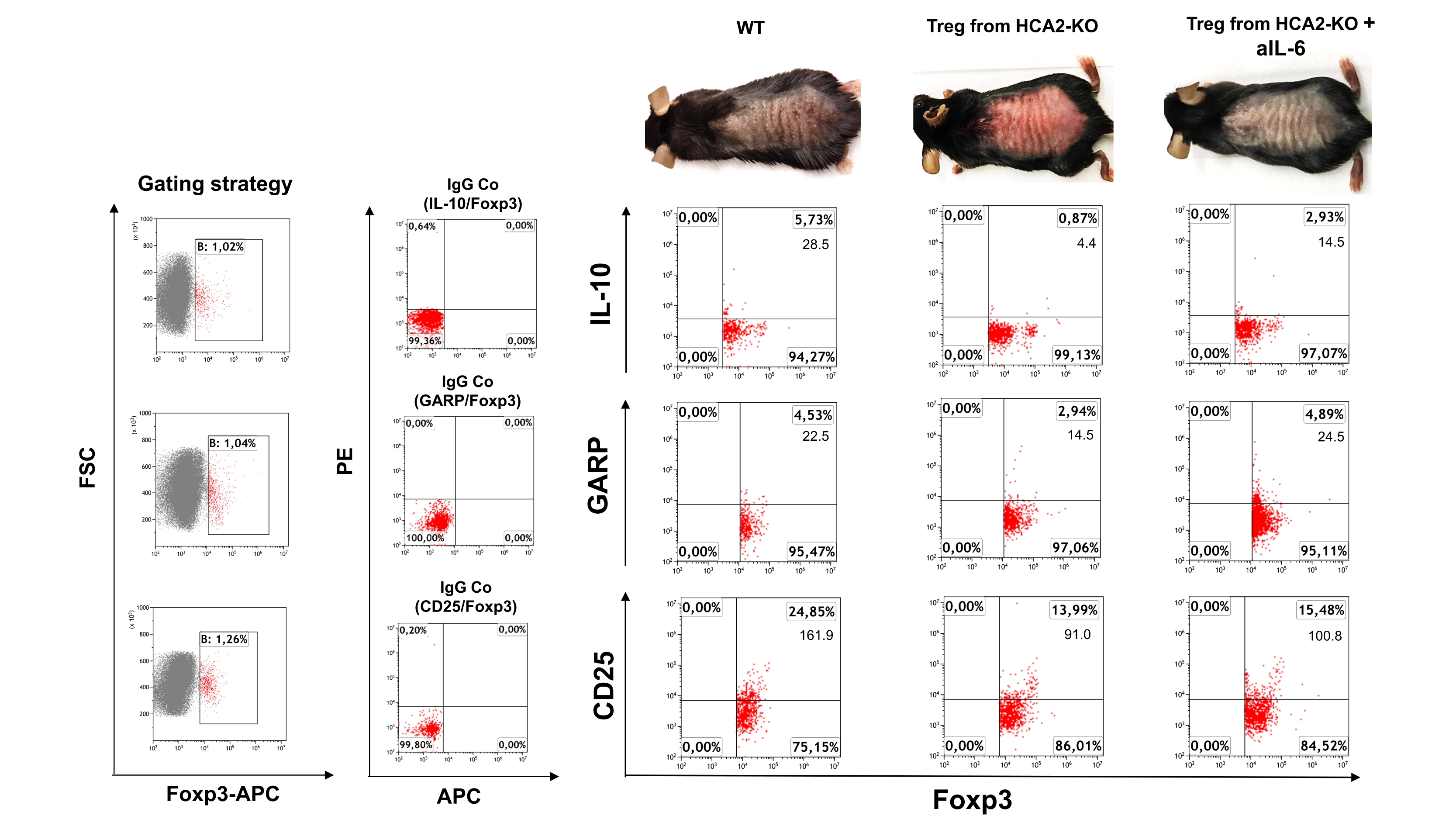

Supplement: Supplementary Figure 5 — Treg were obtained from naïve HCA2-KO or from HCA2-KO mice injected i.p. with a rat monoclonal anti-IL-6 antibody. The isolated Treg were injected i.v. into IMQ-treated WT recipient mice. LN and spleen cells from these recipients were analyzed for double positive Foxp3/IL-10, Foxp3/GARP and Foxp3/CD25 cells and demonstrated as percentage and total number of double positive cells. [file Image_5.jpeg]

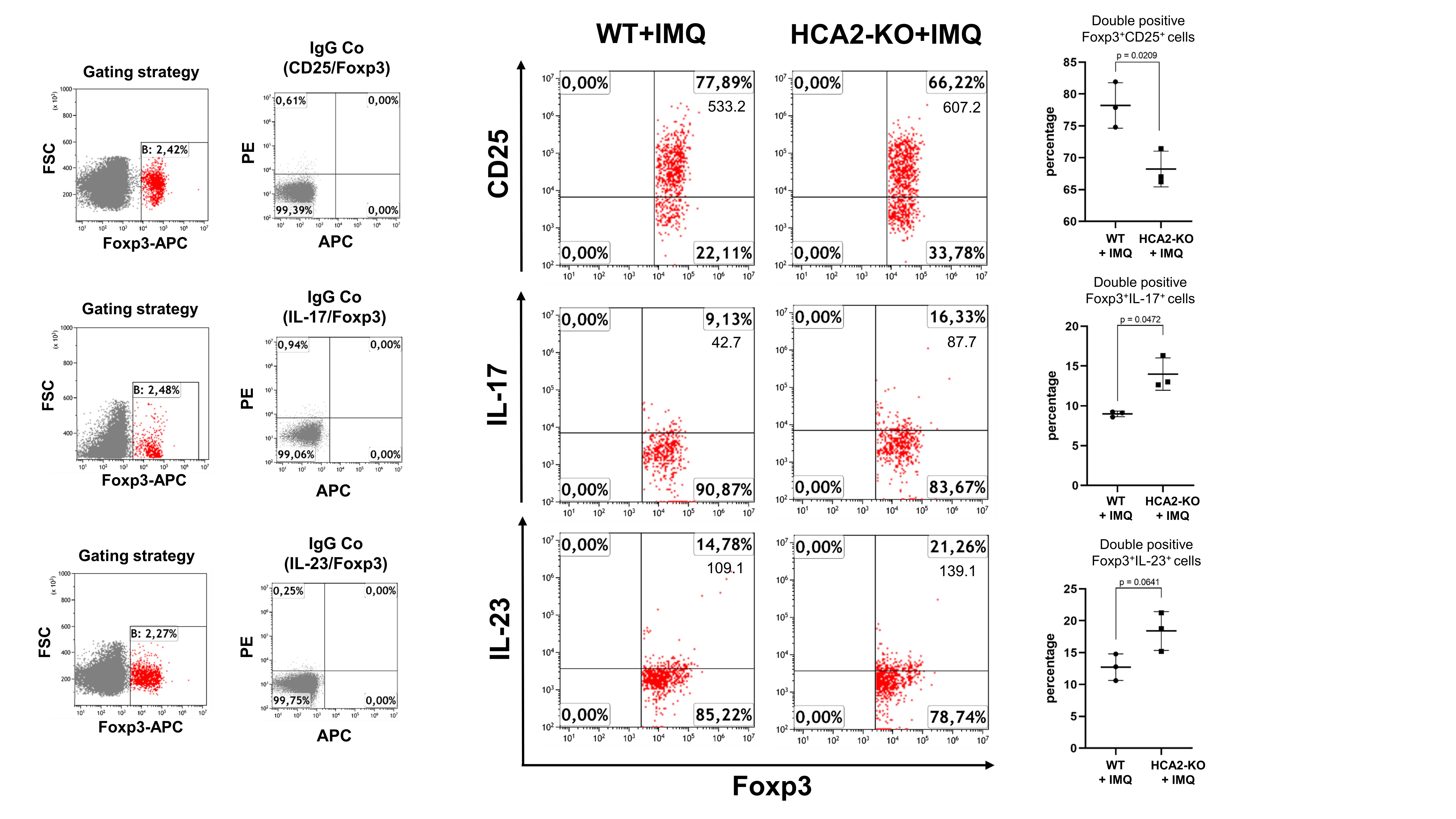

Supplement: Supplementary Figure 6 — Pooled lymph node cells and splenocytes were obtained from 4 IMQ-treated WT and 4 HCA2-KO mice. Cells were gated for Foxp3 and FACS analysis performed for CD25, IL-17 and IL-23. The number of double positive cells is displayed in the histograms. FACS analysis is also shown as scatter graph with mean ± SD. Y axes show percentage of double positive cells. Data were analyzed by using the t test with Welch´s correction (n = 3). [file Image_6.jpeg]
